# Childhood immunizations in China: disparities in health care access in children born to North Korean refugees

**DOI:** 10.1186/s12914-016-0085-z

**Published:** 2016-04-13

**Authors:** Hyun Jung Chung, Seung Hyun Han, Hyerang Kim, Julia L. Finkelstein

**Affiliations:** Cornell University, Ithaca, NY USA; Yonsei University College of Medicine, 50-1 YonseiRo SeodaemunGu, 120-752 Seoul, South Korea; Department of Environment and Health Science, Soonchunhyang University, Asan-si, South Korea; Department of Health Systems and Outcomes, Johns Hopkins University School of Nursing, Baltimore, MD USA

**Keywords:** Immunization, Child health, Health disparities, North Korean refugees in China

## Abstract

**Background:**

Childhood immunization rates are at an all-time high globally, and national data for China suggests close to universal coverage. Refugees from North Korea and their children may have more limited health care access in China due to their legal status. However, there is no data on immunization rates or barriers to coverage in this population.

**Methods:**

This study was conducted to determine the rates and correlates of immunizations in children (≥1 year) born to North Korean refugees in Yanbien, China. Child immunization data was obtained from vaccination cards and caregiver self-report for 7 vaccines and 1:3:3:3:1 series. Age-appropriate vaccination rates of refugee children were compared to Chinese and migrant children using a goodness-of-fit test. Logistic regression was used to determine correlates of immunization coverage for each vaccine and the 1:3:3:3:1 series.

**Results:**

Age-appropriate immunization coverage rates were significantly lower in children born to North Korean refugees (12.1-97.8 %), compared to Chinese (99 %) and migrant (95 %) children. Increased father’s age and having a sibling predicted significantly lower vaccination rates.

**Conclusions:**

Children born to North Korean refugees had significantly lower immunization rates, compared to Chinese or migrant children. Further research is needed to examine barriers of health care access in this high-risk population.

## Background

North Korea has faced famine, severe food shortages, persecution, and human rights violations, and in the late 1990s thousands of North Koreans fled across the border into China [1–3]. The Chinese government classifies North Korean refugees as illegal economic migrants, differentiating them from other refugees [2, 4]. Northeast China has a 14:1 male:female ratio, which drives a demand for North Korean “bride-slaves” sold to Chinese men [5–7]. Children born to North Korean refugee women and Chinese men are not recognized as citizens because of the legal status of their mothers. These “stateless” children are excluded from the public health care system [7, 8]. A “stateless” person is defined in the 1954 Convention relating to the Status of Stateless Persons as “a person who is not considered as a national by any State under the operation of its law”.

Routine childhood vaccinations are among the most cost-effective preventive health services [9]. Childhood immunization rates are at an all-time high worldwide, and national data for China suggests close to universal immunization coverage [10–12]. The Chinese government has strived to exceed the goal of 90 % immunization rates for children. According to the review of surveillance data from the China Center for Disease Control and Prevention, reported immunization rates for all required childhood vaccines has been 99 % in China every year since 2005 [13]. National-level data may conceal disparities in immunization rates, particularly in marginalized and minority sub-populations [9]. For example, in a previous study of immunization status and risk factors among children in Bejing, China, migrant children had significantly lower age-appropriate immunization rates for the 1:3:3:3:1 series, compared to registered Chinese children [14]. Similarly, in a study of immunization coverage and its determinants among children in Zhejiang, China, 54.6 % of migrant children had age-appropriate vaccinations, compared to 92.9 % of Chinese children at 12 months of age [15].

In China, registered children have had access to free vaccinations since the mid-1990s; all vaccines are provided free of charge in public clinics, regardless of insurance status [14]. The Chinese government has developed specific programs to provide vaccinations to migrant children, including house-to-house visits by health care workers to identify incompletely vaccinated children and immunize them in clinics. Children born to North Korean refugee women and Chinese men, however, are not recognized by the government as citizens and are excluded from public health care system [7, 8]. Although there is limited data on this sub-population, it is estimated that 10,000 to 50,000 children are born each year to North Korean refugee women and Chinese men.

Refugees from North Korea and their children may have more limited health care access in China due to their legal status. However, there is no data on the immunization rates or barriers to vaccination coverage in this high-risk population. The objective of this study was to determine the rates of immunization in children born to North Korean refugee women and Chinese men in China, and to identify predictors of vaccination status.

## Methods

### Design

**Fig. 1:**
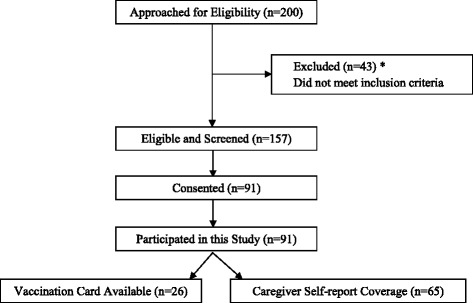
Consort Diagram For Participant Recruitment * Inclusion Criteria : Having a child 12 months of age and older; Born to a North Korean refugee mother (who crossed the North Korean-Chinese border in the past 20 years) and a Chinese man, residence in the Yanbien prefecture; Participation in an NGO (Global Together) program, which provides support for North Korean refugee women and their families

A cross-sectional survey was administered to 91 North Korean refugee caregivers of children over 12 months of age, residing in the Yanbien prefecture in northwest China. A total of 200 North Korean refugees were approached and screened for potential participation in this study; 157 individuals were eligible to participate, and 91 caregivers consented to participate in this study and responded to the questionnaire. A Consort diagram of the participant recruitment is presented in Fig. 1. Immunization rates, and socio-demographic, health, and obstetric history data were collected from all participants. The study protocol was explained in detail to the caregivers, and informed written consent was obtained prior to study enrollment. The study protocol was approved by the Institutional Review Board for human participants at Cornell University, and was also approved by the appropriate local authorities in China with permission letters from independent local community NGO leaders stating that this research is in keeping with local social standards and expectations.

### Participant characteristics

The participants in this study were primary caregivers of children born to North Korean refugee women and Chinese men. A primary caregiver was defined as a parent, a grandparent, or another close relative who was caring for the child at the time of the study. Inclusion criteria were: having a child 12 months of age and older born to a North Korean refugee mother (who crossed the North Korean-Chinese border in the past 20 years) and a Chinese man, residence in the Yanbien prefecture in China, and participation in an NGO (Global Together) program which provides support for North Korean refugee women and their families. The NGO provides services to approximately 20 % of the population of North Korean refugees in this area. This NGO provides services to approximately 20 % of the population of North Korean refugees in this area. Almost all North Korean refugees reach out to local Korean churches in China for assistance and to register their names in this system, and the NGO randomly selected individuals from this population. In addition to random selection of potential participants, there are no major differences between those who participate in the NGO program and those who do not. This age group was selected based on the Ministry of Health guidelines in China that require completion of primary immunizations (1:3:3:3:1 series) by 12 months of age [11]. The vaccination requirements and age-appropriate schedule are presented in Table 1.

**Table 1:**
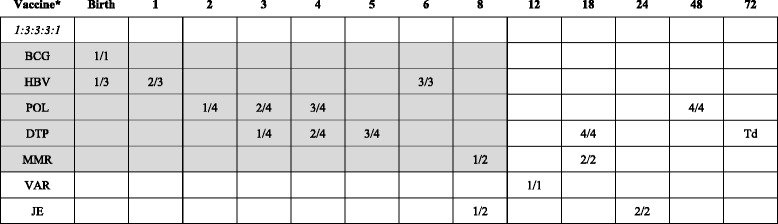
Immunization schedule for Children (2012, WHO, MOH China)

^a^Age in months

*BCG* tuberculosis vaccine; *DTP* diphtheria-tetanus-pertussis vaccine, *HBV* hepatitis B vaccine, *MMR* measles-mumps-rubella vaccine, *POL* polio vaccine, *Td* Tetanus toxoid vaccine

The schedules and vaccines of 1:3:3:3:1 series were represented with shaded box

### Data collection

A structured questionnaire was administered to 91 primary caregivers of Korean-Chinese children from June to August, 2010. This questionnaire comprised 32 closed-ended items, including vaccination status, socio-demographic (e.g., child’s gender, age, place of birth, maternal and paternal educational level, father’s region of origin) data, health (e.g., health condition at birth, current health status) data, obstetric history (e.g., previous history of miscarriage), and origin of birth. The questionnaire was given face-to-face in Yanbien to caregivers by a trained research assistant from the NGO.

### Immunization data

Child immunization data was extracted from vaccination cards or mainly assessed by caregiver self-report, due to the low availability of vaccination card. Among 91 participants, 23 participants had vaccination cards available, and 68 participants depended on caregiver’s written self-reports as presented in Fig. 1. The immunization rates of children with vaccination cards and those without vaccination cards were similar for each of the vaccines (*p* > 0.05) (Table 2). Data for seven vaccines were collected, including the five vaccines as part of the 1:3:3:3:1 series: BCG (tuberculosis), diphtheria-tetanus-pertussis (DTP), hepatitis B (HBV), measles-mumps-rubella (MMR), polio (POL); and two additional vaccines, varicella (VAR) and Japanese encephalitis (JE).

**Table 2:** Child Immunization Rates by Vaccination Card Status

Vaccines	Children Immunized (%) with vaccination card (*n* = 26)	Children Immunized (%) without vaccination card (*n* = 65)	*P*-value
BCG	92.3 (24/26)	100 (65/65)	0.16
DTP	38.5 (10/26)	18.4 (12/65)	0.29
HBV	73.0 (19/26)	60.0 (39/65)	0.31
MMR	76.9 (20/26)	90.8 (59/65)	0.17
POL	19.2 (5/26)	9.23 (6/65)	0.64

*BCG* tuberculosis vaccine; *DTP* diphtheria-tetanus-pertussis vaccine, *HBV* hepatitis B vaccine, *MMR* measles-mumps-rubella vaccine, *POL* polio vaccine, *Td* Tetanus toxoid vaccine

The vaccination schedule for the first 12 months of life recommended by the World Health Organization (WHO) and the Ministry of Health (MOH) in China in 2012 is presented in Table 1. The 1:3:3:3:1 series includes one dose of BCG (at 1 month of age), three doses of HBV (birth, 1 month, and 6 months), three doses of POL (2, 3, and 4 months), three doses of DTP (3, 4, and 5 months), and one dose of MMR (8 months) [15].

Immunization data for local Chinese children were obtained from the publically available WHO national immunization coverage datasets for China. Immunization data for children aged 12 months and older was selected from 2010, to compare vaccination rates to data from local children from the same year and age group [12]. Immunization data for migrant children were obtained from a previous study published by Hu et al. in 2011 [15]. In this study, a household cluster sampling survey was administered to primary caregivers of 2,632 migrant children aged 12 to 35 months in 2010, using in-person interviews [15].

### Data analysis

Child immunization data was categorized as age-appropriate for each vaccine in Table 1, and for the 1:3:3:3:1 series, based on the monthly vaccination schedule (i.e., correct number and timing of all doses). Rates of vaccines were analyzed for each of the seven vaccines, and for the 1:3:3:3:1 series. Age-appropriate vaccination rates for children in the current study were compared to local Chinese children (WHO) [12] and migrant children (Hu et al) [15] using a goodness-of-fit test. Logistic regression was used to determine predictors of immunization status for children for each of the seven vaccines and for the 1:3:3:3:1 series. Variables with univariate *p*-values of less than 0.20 were included in each of the multivariate regression models (*p* < 0.10, *p* < 0.05 models) and retained if their *p*-values were less than 0.05. All statistical analyses were performed using SPSS software, Version 18.0 (SPSS Inc., Chicago, IL, USA).

## Results

The characteristics of the 91 children and their parents in this study are presented in Table 3. A total of 52.7 % (*n* = 48) of children were female; and 81.3 % (*n* = 74) had a parent for a primary caregiver. The average age of mothers was 37.2 (SD: 5.8) years and the average age of fathers was 41.1 (SD: 6.3) years; 39.6 % (*n* = 36) of participants had annual income of less than 3000 Yuan (Approximately 453 USD). A total of 69.2 % (*n* = 63) of fathers were Han in origin, and 30.8 % (*n* = 28) were Chinese Korean (Chosun); 90.1 % (*n* = 82) of fathers had elementary school education level or no formal education.

**Table 3:** Characteristics of the study population (*n* = 91)

Variable		Mean (SD) or n (%)
*Socio-demographic*		
Gender of Child		
	Male	43 (47.3 %)
	Female	48 (52.7 %)
Age (years)		9.5 (3.1)
	<8	13 (14.3 %)
	8-11	51 (56.0 %)
	≥12	27 (29.7 %)
Total number of interracial children		91 (100.0 %)
Birthplace		
	Heilong Jiang	30 (33.0 %)
	Liaoning	17 (29.7 %)
	Jilin	34 (37.3 %)
Primary caregiver		
	Parent	74 (81.3 %)
	Grandparent	4 (4.4 %)
	Other relative	13 (14.3 %)
Mother’s age (years)		37.2 (5.8)
	<40	60 (65.9 %)
	≥41	31 (34.1 %)
Father’s age (years)		41.1 (6.3)
	<40	33 (36.3 %)
	≥41	58 (63.7 %)
Father’s region of origin		
	Han	63 (69.2 %)
	Chinese-Korean (Chosun)	28 (30.8 %)
Father’s educational level		
	No formal education	8 (8.8 %)
	Elementary school	74 (81.3 %)
	Middle school	7 (7.7 %)
	High school	2 (2.2 %)
Annual income (Yuan^a^)		3544.0 (1837.3)
	<3000	36 (39.6 %)
	3000-6000	48 (52.7 %)
	≥6000	7 (7.7 %)
Siblings		
	No	74 (81.3 %)
	Yes	17 (18.6 %)
*Health-related*		
Health condition at birth (Reported by caregiver)		
	Poor	11 (12.0 %)
	Good	80 (88.0 %)
Present health condition (Reported by caregiver)		
	Very poor	1 (1.1 %)
	Poor	4 (4.4 %)
	Good	70 (76.9 %)
	Very good	16 (17.6 %)
Vaccination diary available		
	No	65 (71.4 %)
	Yes	26 (28.6 %)
Maternal history of miscarriage		
	No	78 (85.7 %)
	Yes	13 (14.3 %)

^a^1 US Dollar was equivalent to approximately 6.62 Yuan at time of data collection

**Fig. 2:**
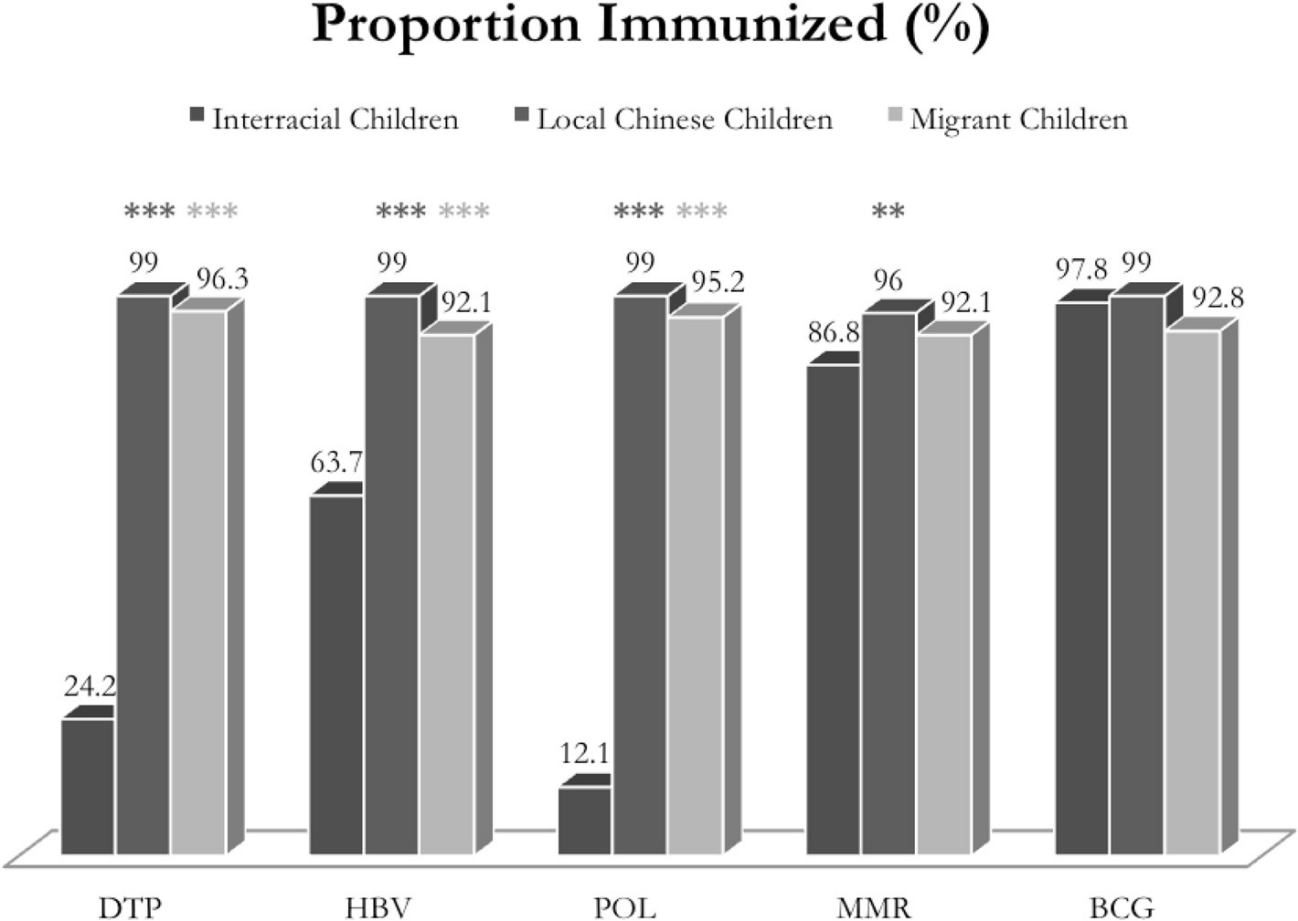
Vaccine Coverage: Immunization Rates of Interracial Children Compared to Local Chinese and Migrant Children. *p* < 0.05 is indicated with *; *p* < 0.01 was marked with **; *p* < 0.001 was marked with ***. * DTP, diphtheria-tetanus-pertussis; HBV, hepatitis B; POL, polio; MMR, measles-mumps-rubella; BCG, tuberculosis

Immunization rates for Korean-Chinese, local Chinese, and migrant children are presented in Table 4. Immunization rates for Korean-Chinese children were significantly lower than for local Chinese children, including DTP (24.2 % vs. 99.0 %; *p* < 0.001), HBV (63.7 % vs. 99.0 %; *P* < 0.001), MMR (86.8 % vs. 96.0 %; *P* = 0.005), and POL (12.1 % vs. 99.0 %; *p* < 0.001) were significantly lower in the Korean-Chinese children, compared to local Chinese children. The rates of BCG were not significantly different (97.8 % vs. 99.0 %; *p* = 0.22). Immunization rates for Korean-Chinese children were also significantly lower than migrant children, including DTP (24.2 % vs. 96.3 %; *p* < 0.001), HBV (63.7 % vs. 92.1 %; *p* < 0.001), and POL (95.2 % vs. 12.1 %; *p* < 0.001) (Table 4). The immunization rates for the 1:3:3:3:1 series for Korean-Chinese children (14.3 %) were significantly lower than for local Chinese children (92.8 %, *p* < 0.001) and migrant children (54.6 %, *p* < 0.001). The immunization rates for Korean-Chinese, Chinese, and migrant children are presented for each vaccine in Fig. 2. The immunization rates of children with vaccination cards and those without vaccination cards were similar for each of the vaccines (*p* > 0.05) (Table 2).

**Table 4:** Vaccine Coverage: comparing immunization rates of interracial children to local Chinese and migrant children

	Children Immunized (%)					Children Immunized (%)				
	Local Chinese	Interracial	Z	*P*-value	Upper conf bound	Migrant	Interracial	Z	*P*-value	Upper conf bound
Vaccines^b^	(*n* = 1,341,335,000)	(*n* = 91)				(*n* = 2,632)	(*n* = 91)			
BCG	99.0 %	97.8 (89)	−0.78	0.218	0.013	92.8 %	97.8 (89)	3.253	0.999	0.075
DTP	99.0 %	24.2 (22)^b^	−16.6	<0.001	−0.674	96.3 %	24.2 (22)^b^	−16	<0.001	−0.647
HBV	99.0 %	63.7 (58)^b^	−7.00	<0.001	−0.270	92.1 %	63.7 (58)^b^	−5.63	<0.001	−0.201
MMR	96.0 %	86.8 (79)^b^	−2.59	0.005	−0.034	92.1 %	86.8 (79)	−1.49	0.068	0.006
POL	99.0 %	12.1 (11)^b^	−25.40	<0.001	−0.813	95.2 %	12.1 (11)^b^	−24.3	<0.001	−0.775

^b^ *BCG* tuberculosis, *DTP* diphtheria-tetanus-pertussis, *HBV* hepatitis B, *MMR* measles-mumps-rubella, *POL* polio

The numbers in parentheses in the column for Interracial Children represent the number of children receiving the full series of each vaccine

The univariate and multivariate correlates of immunization rates for Korean-Chinese children for each of the seven vaccines and for the 1:3:3:3:1 series are presented in Table 5. Having at least one sibling (OR: 0.16, 95 % CI: 0.03-0.74, *p* < 0.05) was associated with significantly lower odds of being vaccinated for HBV. Greater paternal age (>40) was associated with significantly lower odds of being vaccinated for VAR (OR: 0.10, 95 % CI: 0.01-0.95, *p* < 0.05). Also, greater paternal age (>40) was associated with significantly lower odds of being vaccinated for JE (OR: 0.41, 95 % CI: 0.16-1.01, *p* < 0.05). Maternal history of miscarriage was associated with increased likelihood of having POL vaccine (OR: 12.51, 95 % CI: 3.03-51.66, *p* < 0.05) and increased likelihood of having the complete 1:3:3:3:1 series (OR: 7.11, 95 % CI: 2.01-25.13, *p* < 0.05) (Table 5).

**Table 5:** Univariate and multivariate predictors of vaccination coverage

	Univariate (*P* < 0.20)			Multivariate (*P* < 0.10 Model)			Multivariate (*P* < 0.05 Model)		
	OR	95 % CI	*P*-value	OR	95 % CI	*P*-value	OR	95 % CI	*P*-value
Vaccines									
DTP									
Child sex									
Female	Ref								
Male	0.91	(0.35, 2.38)	0.909						
Father’s age									
< 40	Ref								
≥ 41	1.67	(0.63, 4.43)	0.306						
Father’s region of origin									
Han	Ref			Ref					
Chinse-Korean (Chosun)	3.60	(0.97, 13.40)	0.056	3.98	(0.96, 16.39)	0.056			
Father’s educational level									
Middle-High	Ref								
Elementary	2.67	(0.19, 36.76)	0.464						
None	2.76	(0.32, 23.57)	0.353						
Annual income									
< 3000	Ref								
≥ 3000	1.38	(0.52, 3.67)	0.517						
Sibling									
No	Ref			Ref					
Yes	6.34	(0.79, 50.87)	0.082	5.63	(0.67, 47.28)	0.111			
Vaccination card									
No	Ref			Ref					
Yes	2.76	(1.01, 7.57)	0.048	1.70	(0.56, 5.11)	0.349			
Previous miscarriage									
Yes	Ref			Ref					
No	0.30	(0.89, 1.02)	0.054	0.35	(0.09, 1.38)	0.124			
HBV									
Child sex									
Female	Ref								
Male	0.93	(0.39, 2.18)	0.859						
Father’s age									
< 40	Ref								
≥ 41	0.99	(0.41, 2.42)	0.988						
Father’s region of origin									
Han	Ref			Ref					
Chinse-Korean (Chosun)	2.32	(0.93, 5.79)	0.072	2.55	(0.95, 7.74)	0.067			
Father’s educational level									
Middle-High	Ref			Ref			Ref		
Elementary	0.38	(0.03, 5.17)	0.464	0.20	(0.01, 3.01)	0.243	0.28	(0.02, 3.95)	0.343
None	0.18	(0.02, 1.54)	0.119	0.11	(0.01, 1.00)	0.050	0.14	(0.02, 1.17)	0.069
Annual income									
< 3000	Ref								
≥ 3000	0.68	(0.29, 1.63)	0.387						
Sibling									
No	Ref			Ref			Ref		
Yes	0.19	(0.04, 0.87)	0.032	0.16	(0.03, 0.78)	0.021	0.16	(0.03, 0.74)	0.019
Vaccination card									
No	Ref								
Yes	0.84	(0.43, 2.87)	0.836						
Previous miscarriage									
Yes	Ref								
No	1.12	(0.33, 3.74)	0.859						
MMR									
Child sex									
Female	Ref								
Male	0.88	(0.26, 2.97)	0.838						
Father’s age									
< 40	Ref								
≥ 41	0.77	(0.22, 2.65)	0.677						
Father’s region of origin									
Han	Ref								
Chinse-Korean (Chosun)	1.15	(0.32, 4.17)	0.836						
Father’s educational level									
Middle-High	Ref			Ref					
Elementary	3.50	(0.28, 43.16)	0.328	3.90	(0.30, 50.59)	0.298			
None	4.13	(0.86, 19.79)	0.077	3.50	(0.70, 17.60)	0.128			
Annual income									
< 3000	Ref								
≥ 3000	0.90	(0.26, 3.10)	0.873						
Sibling									
No	Ref								
Yes	1.55	(0.37, 6.46)	0.549						
Vaccination card									
No	Ref			Ref					
Yes	0.34	(0.10, 1.17)	0.087	0.37	(0.10, 1.33)	0.127			
Previous miscarriage									
Yes	Ref								
No	2.30	(0.53, 9.96)	0.265						
POL									
Child sex									
Female	Ref			Ref					
Male	0.21	(0.04, 1.04)	0.056	0.23	(0.04, 1.34)	0.101			
Father’s age									
< 40	Ref			Ref					
≥ 41	2.36	(0.66, 8.42)	0.188	2.49	(0.49, 12.69)	0.272			
Father’s region of origin									
Han	Ref								
Chinse-Korean (Chosun)	2.17	(0.44, 10.75)	0.344						
Father’s educational level									
Middle-High	Ref			Ref					
Elementary	0.29	(0.02, 3.52)	0.328	0.51	(0.02, 11.93)	0.674			
None	0.21	(0.04, 1.02)	0.054	0.33	(0.04, 3.08)	0.327			
Annual income									
< 3000	Ref								
≥ 3000	1.32	(0.37, 4.69)	0.671						
Sibling									
No	Ref								
Yes	-	-	-						
Vaccination card									
No	Ref			Ref					
Yes	2.34	(0.65, 8.48)	0.195	1.43	(0.28, 7.33)	0.669			
Previous miscarriage									
Yes	Ref			Ref			Ref		
No	0.08	(0.02, 0.33)	<0.001	0.11	(0.02, 0.51)	0.005	0.08	(0.02, 0.33)	<0.001
VAR									
Child sex									
Female	Ref			Ref					
Male	2.93	(0.56, 15.36)	0.204	1.87	(0.32, 1.99)	0.488			
Father’s age									
< 40	Ref			Ref			Ref		
≥ 41	0.31	(0.07, 1.37)	0.122	0.10	(0.01, 0.95)	0.045	0.10	(0.01, 0.95)	0.045
Father’s region of origin									
Han	Ref								
Chinse-Korean (Chosun)	1.40	(0.31, 6.30)	0.667						
Father’s educational level									
Middle-High	Ref			Ref			Ref		
Elementary	-	-	-	-	-	-	-	-	-
None	6.90	(1.32, 36.17)	0.022	18.55	(1.67, 205.8)	0.017	21.54	(2.01, 231.3)	0.011
Annual income									
< 3000	Ref								
≥ 3000	1.10	(0.25, 4.92)	0.901						
Sibling									
No	Ref								
Yes	0.60	(0.07, 5.21)	0.642						
Vaccination card									
No	Ref								
Yes	0.64	(0.14, 2.89)	0.561						
Previous miscarriage									
Yes	Ref								
No	2.18	(0.39, 12.20)	0.374						
JE									
Child sex									
Female	Ref			Ref					
Male	2.10	(0.90, 4.87)	0.085	2.51	(0.98, 6.43)	0.054			
Father’s age									
< 40	Ref			Ref			Ref		
≥ 41	0.41	(0.16, 1.00)	0.050	0.36	(0.14, 0.97)	0.044	0.41	(0.16, 1.01)	0.050
Father’s region of origin									
Han	Ref								
Chinse-Korean (Chosun)	0.87	(0.35, 2.12)	0.751						
Father’s educational level									
Middle-High	Ref								
Elementary	2.00	(0.28, 14.20)	0.488						
None	1.61	(0.37, 6.93)	0.523						
Annual income									
< 3000	Ref			Ref					
≥ 3000	1.81	(0.77, 4.24)	0.172	2.34	(0.91, 6.05)	0.081			
Sibling									
No	Ref			Ref					
Yes	0.35	(0.12, 1.06)	0.062	0.30	(0.09, 1.04)	0.057			
Vaccination card									
No	Ref			Ref					
Yes	0.36	(0.13, 0.97)	0.042	0.42	(0.14, 1.25)	0.119			
Previous miscarriage									
Yes	Ref								
No	1.93	(0.55, 6.79)	0.307						
1:3:3:3:1									
Child sex									
Female	Ref			Ref					
Male	0.49	(0.17, 1.44)	0.192	0.37	(0.18, 1.89)	0.370			
Father’s age									
< 40	Ref								
≥ 41	1.54	(0.54, 4.38)	0.422						
Father’s region of origin									
Han	Ref								
Chinse-Korean (Chosun)	1.12	(0.38, 3.75)	0.759						
Father’s educational level									
Middle-High	Ref			Ref					
Elementary	0.18	(0.02, 2.12)	0.173	0.31	(0.02, 4.81)	0.404			
None	0.27	(0.06, 1.13)	0.073	0.45	(0.09, 2.28)	0.337			
Annual income									
< 3000	Ref								
≥ 3000	0.72	(0.24, 2.12)	0.547						
Sibling									
No	Ref			Ref					
Yes	4.77	(0.59, 38.64)	0.143	2.76	(0.32, 23.73)	0.356			
Vaccination card									
No	Ref								
Yes	1.81	(0.61, 5.34)	0.283						
Previous miscarriage									
Yes	Ref			Ref			Ref		
No	0.14	(0.04, 0.50)	0.002	0.18	(0.05, 0.69)	0.012	0.14	(0.04, 0.50)	0.002

* - indicates “not able to be calculated”

*DTP* diphtheria-tetanus-pertussis, *HBV* hepatitis B, *MMR* measles-mumps-rubella, *POL* polio, *VAR* varicella zoster, *JE* Japanese encephalitis, *1:3:3:3:1* age-appropriate 1 dose of tuberculosis, 3 doses of hepatitis, 3 doses of diphtheria-tetanus-pertussis, 3 doses of polio virus and 1 dose of measles-mumps-rubella vaccines series, *Ref* reference

## Discussion

### Main findings of this study

In this study, children born to North Korean refugee women and Chinese men had significantly lower rates of immunizations, compared to Chinese or migrant children. Korean-Chinese children had significantly lower immunization rates for diphtheria-tetanus-pertussis, hepatitis B, measles-mumps-rubella, and polio compared to locally registered Chinese children; and lower rates of immunization for diphtheria-tetanus-pertussis, hepatitis B, and polio, compared to migrant children. In contrast, rates of single-dose BCG were similarly high in all children. Increased paternal age and having a sibling were associated with lower odds of being vaccinated, while maternal history of miscarriage was associated with increased likelihood of children being vaccinated.

### What is already known on this topic

Vaccines are the most cost-effective interventions for child survival, and access to essential childhood vaccinations is considered a human right. [9, 16] Childhood immunization rates are at an all-time high worldwide, and national data for China suggests close to universal immunization coverage [10–12]. However, overall global and national-level immunization rate data may conceal disparities in vaccination rates at sub-national levels [17], particularly in marginalized and minority sub-populations [9].

Low rates of immunization coverage have been reported in previous studies in China among migrant children [14, 15] and in other resource-limited settings in South-East Asia and Sub-Saharan Africa [17]. In a previous study among children in Zhejiang, China, vaccination rates were significantly lower in migrant children, compared to local Chinese children [15]. At eight months of age, 42.2 % of migrant children were age-appropriately vaccinated for the 1:3:3:3:1 series, compared to 69.7 % of local Chinese children. This gap in vaccination coverage increased by 12 months of age: only 54.6 % of migrant children completed age-appropriate vaccinations for the 1:3:3:3:1 series by 12 months, compared to 92.9 % of local Chinese children [15]. Also, in a research study conducted among migrant children in Beijing, China, migrant children had significantly lower immunization rates compared to registered Chinese children (65 % vs. 96 %); authors concluded that interventions were needed for migrant children to achieve universal immunization coverage.

In studies examining barriers to immunization coverage in children, parental educational level [18, 19], number of siblings in household [20], other socioeconomic factors [21–23], distance and cost [22] have been identified as barriers. Other studies have identified parental perception, health beliefs, and attitudes toward child immunizations as important predictors of completion of age-appropriate immunizations [24, 25]. Also, in a research study conducted among migrant children in Beijing, China, primary caregiver’s age, educational level, and awareness of the importance of immunization were important predictors of immunization status [14].

### What this study adds

This is the first study to examine the rates of immunization in children born to North Korean refugee women and Chinese men. Additionally, this study identified correlates and potential barriers to vaccination status. Korean-Chinese children had significantly lower vaccination rates, compared to local registered Chinese children or migrant children. Findings indicate that health disparities exist in children born to North Korean refugees and Chinese men, and suggest that this may be a high-risk population, which warrants further investigation.

In the present study, increased father’s age was associated with significantly lower likelihood of being vaccinated. This is consistent with findings from a previous study among migrant children in Bejing, China [14]. Other studies have found that increased parental education [18] is associated with increased likelihood of childhood immunizations. Together, findings suggest paternal sociodemographic factors may be an important correlate of child vaccination status. Findings are also consistent with previous studies that found that increased number of siblings in a family was associated with increased odds of not being vaccinated [19].

In this study, maternal history of miscarriage was associated with increased likelihood of children being vaccinated. Although this has not been observed in previous studies, other studies have identified parental perception, health beliefs, and attitudes toward child immunizations are important predictors of completion of age-appropriate immunizations [24, 25]. It is possible that a previous miscarriage may influence parental attitudes, beliefs regarding health services, perceptions toward child immunizations, or health-seeking behaviors.

### Limitation of this study

This study has a few limitations. The cross-sectional study design and retrospective assessment of vaccination rates and barriers to coverage are study limitations. Also, assessment of vaccination coverage was based on data extracted from vaccination cards, or caregiver self-reported coverage, for children for whom there was no vaccination card available. The low availability (25 %) of vaccination cards and heterogeneous data sources remains a study limitation. Additionally, although this is the largest study to date to examine vaccination rates and barriers in this population, the relatively small sample size of 91 caregivers and children and limited number of predictors assessed for vaccination coverage are study limitations. Immunization rates for Korean-Chinese children were also compared to local Chinese and migrant children, using historic datasets from WHO [12] and Hu et al.; [15] the heterogeneous data sources limits comparability of findings. Further research is needed to examine the rates and predictors of childhood vaccinations prospectively in this high-risk refugee population.

## Conclusions

In this study, vaccination rates were significantly lower in children born to North Korean refugees and Chinese men, compared to local Chinese or migrant children. Socio-demographic factors, including older paternal age and having a sibling were associated with lower odds of being vaccinated, while maternal history of miscarriage was associated with greater likelihood of being vaccinated. Additional prospective studies are needed to examine rates of immunizations and the barriers and determinants of health care access in this high-risk refugee population.

## Abbreviations

BCG: tuberculosis
DTP: diphtheria-tetanus-pertussis
HBV: hepatitis B
JE: Japanese Encephalitis
MMR: measles-mumps-rubella
MOH: Ministry of Health
NGO: non-governmental organization
POL: polio
VAR: varicella
WHO: World Health Organization
